# The Effect of Salivary pH on the Flexural Strength and Surface Properties of CAD/CAM Denture Base Materials

**DOI:** 10.1055/s-0042-1749160

**Published:** 2022-07-12

**Authors:** Maryam Alzaid, Fatemah AlToraibily, Faisal D. Al-Qarni, Ahmad M. Al-Thobity, Sultan Akhtar, Saqib Ali, Fahad A. Al-Harbi, Mohammed M. Gad

**Affiliations:** 1 College of Dentistry, Imam Abdulrahman Bin Faisal University, Dammam, Saudi Arabia; 2 Department of Substitutive Dental Sciences, College of Dentistry, Imam Abdulrahman Bin Faisal University, Dammam, Saudi Arabia; 3 Department of Biophysics, Institute for Research and Medical Consultations (IRMC), Imam Abdulrahman Bin Faisal University, Dammam, Saudi Arabia; 4 Department of Biomedical Dental Sciences, College of Dentistry, Imam Abdulrahman Bin Faisal University, Dammam, Saudi Arabia

**Keywords:** CAD-CAM, 3D printing, polymethyl methacrylate denture base, saliva, mechanical test

## Abstract

**Objectives** This study aimed to evaluate the influence of different salivary pH on flexural strength, hardness, and surface roughness of computer-aided design and computer-aided manufacturing (CAD/CAM) milled and three-dimensional (3D)-printed denture base resins.

**Methods** One heat-polymerized, two CAD/CAM milled (IvoCad, AvaDent), and two 3D-printed (FormLabs, NextDent) denture base resins were fabricated and divided into five groups ( *n* = 10) according to the solutions: three groups were immersed in different salivary pH (5.7, 7.0, or 8.3), one group was immersed in distilled water (DW) as a positive control, and one group had no immersion (negative control). All immersions were performed at 37°C for 90 days. Flexural strength, hardness, and surface roughness were measured before and after immersion. Data was analyzed with analysis of variance and post hoc Tukey's test ( *α* = 0.05).

**Results** After immersion, all specimens had lower flexural strength values when compared with those with no immersion. Comparing the immersion groups, the highest flexural strength value (93.96 ± 3.18 MPa) was recorded with IvoCad after immersion in DW while the lowest value (60.43 ± 2.66 MPa) was recorded with NextDent after being immersed in 7.0 pH saliva. All specimens had significant decrease in hardness except IvoCad and AvaDent specimens where both presented the highest surface hardness (53.76 ± 1.60 Vickers hardness number [VHN]) after immersion in DW while NextDent showed the lowest hardness value (24.91 ± 2.13 VHN) after being immersed in 8.3 pH saliva. There was statistically significant difference between the baseline and different artificial salivary pH solutions in terms of surfaces roughness, with the highest surface roughness were found in 3D-printed resin materials.

**Conclusion** After exposure to artificial saliva with different salivary pH, the milled CAD/CAM denture base resins showed higher flexural strength, hardness, and lesser surface roughness than conventional and 3D-printed denture base resins.

## Introduction

Poly(methyl methacrylate) (PMMA) is a popular material used to construct dentures. 1 It is frequently used for its multiple advantages including biocompatibility, low cost, and easily processed with fair esthetics characteristics. 2 However, it has low mechanical and physical properties. 1 3 Denture base material is influenced by different factors, as prior studies have shown. 4 5 6 It was reported that the changes of water bath temperature at 40°C and 100°C revealed substantial alterations in surface hardness. 4 Water sorption can also influence denture base resin during polymerization, as water molecules interpose with polymeric chains, they modify the physical properties of the consequent polymer, which could result in extreme dimensional instability. 5

Acidic contents of food and beverages may accelerate the deterioration of chemical structures on the resin surface, which may increase the level of surface roughness on the acrylic resin leading to cracks and low resistance to fractures. 6 Acids react chemically with acrylic resin by filling gaps that exist among polymer chains, causing polymer chains separation. 6 An example is acetic acid, which has been reported to deteriorate the bonding of acrylic resin polymers. 6

During daily life, possible changes in dentures occurred since the oral pH could be reduced by different conditions such as sugar consumption that could allow bacterial growth and acid production. 7 Another possible condition is related to the gastroesophageal reflux where the gastric contents could be transferred spontaneously to the esophagus and oral cavity leading to a remarkable decrease in the oral pH. In addition, it has been found that patients having medical conditions related to hyposalivation or drug-induced xerostomia are most exposed to acidic oral pH due to the reduction of the saliva production and subsequently reduction in the buffering capacity. Furthermore, the overgrowth of microorganism intraorally could be induced and the oral environment would be more acidic. 8 Salivary pH could sometimes be acidic as a result of the consumption of different kinds of foods such as sugar, orange juice, and pastries, or due to smoking, low salivary flow secondary to Sjögren's syndrome, or chemotherapy. 9 On the other hand, salivary pH might be alkaline because of food as amaranth or digestive disorders. 9 Constantinescu et al stated that denture base acrylic resins displayed an increase in surface roughness as the salivary pH became more acidic. 10 Another study showed that variation in salivary pH may affect the adaptation of denture bases and to underlying tissues. 9

With recent developments in digital dentistry, construction of dentures using computer-aided design and computer-aided manufacturing (CAD/CAM) has been more prevalent. 11 There are two techniques to construct dentures with CAD/CAM technology; subtractive milling or additive three-dimensional (3D) printing. 12 13 Comparing the CAD/CAM techniques with the conventional ones, CAD/CAM showed improved mechanical properties, adaptability, with similar biocompatibility, and better patient satisfaction due to a decrease in patient clinical visits. 12 14 Moreover, 3D printing has some advantages, in addition to its wide distribution and popularity, economical as no waste materials can be produced and permits the concurrent manufacturing of multiple products. 15 16

Dentures are always in contact with saliva while in the oral cavity, and studies investigating the influence of saliva on denture base resins are limited. Moreover, the influence of saliva on dentures made with CAD/CAM technology has never been evaluated. Therefore, this study was designed to investigate the influence of various artificial saliva with different pH on the flexural strength and surface properties of both milled and 3D-printed denture base materials, in comparison to conventionally heat-processed resin. The null hypothesis was that different artificial salivary pH would have no influence on flexural strength, hardness, and surface roughness of milled and 3D-printed denture base resins.

## Materials and Methods

A power analysis was performed to calculate the sample size. The formula of the World Health Organization was applied using 0.05 of level of significance and 80% power, and it showed that 10 specimens per group is sufficient to provide reliable results. Specimens were prepared based following International Organization for Standardization (ISO) 20795–1:2013 standards. 17 Flexural strength specimens ( *n* = 250) were made in dimensions of 64 × 10 × 3.3 ± 0.2 mm, while hardness and surface roughness (Ra) ( *n* = 250) rectangular with dimension (15 × 2 mm) has been fabricated. A total of 500 specimens were fabricated. The fabrication and polymerization procedures, materials composition, and manufacturers are summarized in Table 1 . Each group was further divided into subgroups ( *n* = 10) according to the following immersion solutions: artificial saliva with acidic pH = 5.7, artificial saliva with neutral pH = 7.0, artificial saliva with basic pH = 8.3, and distilled water (DW). One group was not placed in any solution to act as a negative control.

**Table 1:** Details of materials used in the present study and fabrication method

**Material**	**Brand name**	**Composition**	**Preparation and polymerization**
Conventional heat-polymerized PMMA	Heat-polymerized acrylic resin (Major Base.20, Major Prodotti Dentari Spa, Momcalieri, Italy)	Powder: Polymer (PMMA) + initiator (benzoyl peroxide [BPO]) (0.5%) + pigments (salts of cadmium or iron or organic dyes) Liquid: Monomer (MMA) + cross-linking agent (Ethylene glycol dimethacrylate [EGDMA] 10%) + inhibitor (hydroquinone)	Polymerization cycle: 90 minutes in a water bath by heating to 74°C, then 100°C for 30 minutes
Pre-polymerized PMMA block	IvoCad (Ivoclar Vivadent, Schaan, Liechtenstein)	Prepolymerized PMMA discs 50–100% methyl methacrylate 2.5–10% 1,4-butanediol dimethacrylate	Discs were cut to the required dimension using diamond saw (Isomet 5000 Linear Precision Saw, Buehler Ltd, Bluff, IL)
	AvaDent (AvaDent Digital Dental Solutions, Scottsdale, AZ, USA)	Prepolymerized PMMA (PMMA 99.5%, pigments < 1.0%)	
3D-printed resin	NextDent Denture 3D+ (NextDent, Vertex-Dental B.V, Soesterberg, The Netherlands)	Ethoxylated bisphenol A dimethacrylate 7,7,9 (or 7,9,9)-trimethyl-4,13-dioxo-3,14-dioxa-5,12- diazahexadecane-1,16-diyl bismethacrylate 2-hydroxyethyl methacrylate silicon dioxide diphenyl(2,4,6- trimethylbenzoyl)phosphine oxide titanium dioxide	Printer: NextDent 5100 (Vertex-Dental B.V, Soesterberg, The Netherlands) Orientation: 90° Layer thickness: 50 μm Cleaning: Isopropyl alcohol 99.9%, Saudi Pharmaceutical Industries, Riyadh, KSA) Post curing time: 15 minutes/80°
	FormLabs denture base LP (FormLabs Inc, Somerville, MA, USA)	55–75% w/w urethane dimethacrylate, 15–25% w/w methacrylate monomers, and < 0.9% w/w phenyl bis(2,4,6-trimethylbenzoyl)-phosphine oxide	Printer: FormLabs 2 (FormLabs Inc, Somerville, MA, USA) Orientation: 90° Layer thickness: 50 μm Cleaning: Isopropyl alcohol 99.9%, Saudi Pharmaceutical Industries, Riyadh, KSA) Post curing time: 15 minutes/80°

Abbreviations: 3D, three-dimensional; PMMA, poly(methyl methacrylate).

### Specimens' Preparation

#### Fabrication of Conventionally Processed Specimens

Specimens were prepared using compression mold technique miming laboratory procedures for denture fabrication. The wax specimens were prepared and invested in hard dental stone within metal flask. Once the stone is completely set, all wax are melted away to create mold spaces using wax elimination machine, then all stone surface was rinsed with hot water to remove wax flashes. While the stone still warm, separating medium was applied to all stone surfaces in two layers. Heat-polymerized acrylic resin (Major base 20, Major Prodotti Dentari, SPA, Italy) was mixed, packed at dough stage, pressed using pneumatic press (1,250 kgf load for 5 minutes), and then left aside for 30 minutes before processing. The flask with specimens was placed into curing unit and processed for 90 minutes in a water bath by heating to 74°C, and then to 100°C for 30 minutes. 18

#### Fabrication of CAD/CAM Specimens

Acrylic resin discs of two different materials (IvoCad, AvaDent) have been sectioned to the required dimensions using Isomat saw under water coolant (Isomet 5000 Linear Precision Saw, Buehler Ltd, Bluff, Illinois, United States). 13 For 3D-printed specimens' design, an open-source CAD software program was used (123D Design, Autodesk, version 2.2.14, California, United States). Design was stored as Standard Tessellation Language files and exported to 3D-printing software. A shaker was used for homogenous composition of printed resin within the containers for 15 minutes. The fluid resins were placed in resin tanks and specimens were printed following manufacturers' recommendations ( Table 1 ), followed by removal of support extensions.

#### Specimens Finishing and Polishing

Finishing of polymerized specimens were performed using silicon carbide grinding papers (800, 1,500, and 2,000 grit) 15 with an abundant amount of water, followed by standardized polishing using a rag wheel and pumice (materials specs). The specimen's dimensions were measured for verification using digital caliber followed by storage in DW for 72 hours at 37°C. 18

### Preparation of Artificial Saliva

Fresh artificial saliva solution was formulated by mixing NaCl 0.400 g, KCl 0.400 g, NaH _2_ PO _4_ .H _2_ O 0.69 g, CaCl _2_ .H _2_ O 0.795 g, and Na _2_ S.9H _2_ O 0.005 g in 1,000 mL of deionized water (as proposed by Fusayama et al). 19 The pH of freshly synthesized saliva was 5.3 to 5.5. The pH was then adjusted to the three desired pH values, that is, 5.7, 7, and 8.3 by adding aliquots of 0.25 mL of NaOH (as proposed by Fusayama et al 19 and Farooq et al, 20 ), until they reached the desired pH values. For each pH value, a separate glass container was used with a plastic lid.

### Immersion Protocol

All samples were stored in artificial saliva of different pH (5.7, 7, or 8.3) at 37°C for 16 hours, and for 8 hours in DW to simulate the daily patient use of dentures. Samples were held in containers by dental floss to ensure that all samples were surrounded by the solutions in all aspects. Immersion solutions were replaced weekly. This cycle was repeated for each specimen for 90 days 9 and a pH meter was used weekly to ensure that the salivary pH is maintained as required, that is, 5.7, 7, and 8.3. All specimens were tested for flexural strength, hardness, and surface roughness before (T _0_ ) and after 90 days' immersion.

### Testing Procedure

Flexural strength was evaluated with a three-point bending test using a universal testing machine (Instron 8871; Instron Co., Norwood, Massachusetts, United States). The load (5-KN cell force) was applied at the middle of the specimens with equal distance from two supports with crosshead speed of 5 mm/min. Fracture load was recorded at failure to calculate flexural strength according the ISO recommendation 17 and as described previously. 15

**Fig. 1:**
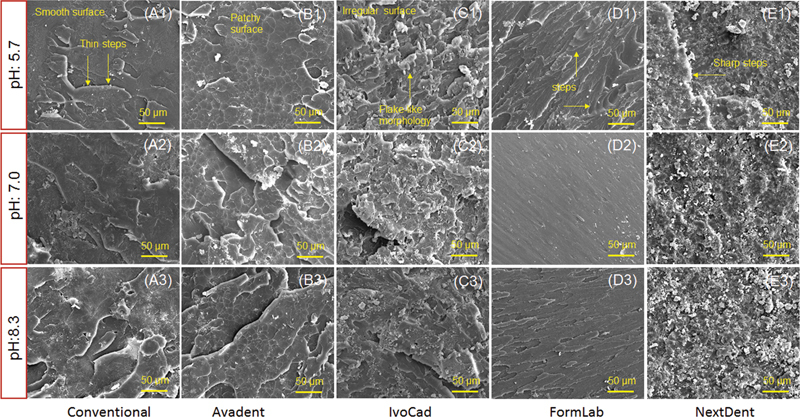
Representative scanning electron microscope (SEM) micrographs of fractured surfaces of different denture base reins; (A1–A3) heat polymerized, (B1–B3) AvaDent, (C1–C3) IvoCad, (D1–D3) FormLabs, (E1–E3) NextDent under three pH values (5.7, 7.0, and 8.3). The scale bars are 50 µm.

Scanning electron microscope (SEM; Field Electron and Ion Company, Inspect S50, Czech Republic at 20 kV) was utilized to investigate the surface morphology of heat-polymerized, two milled (IvoCad and AvaDent), and two 3D-printed (FormLabs and NextDent) denture base resins (total 5 specimens for SEM) under three pH values (5.7, 7.0, and 8.3 pH). SEM micrographs were displayed at representative magnification of ×1,000 (with scale bar of 50 µm) for all the specimens to highlight surface features ( Fig. 1 ). The brittle fracture mode is defined as irregular surface with sharp steps, while ductile fracture modes are represented by smooth background with mirror-like appearance. Intermediate fracture modes indicate fractures that are in-between brittle and ductile modes.

For hardness measurement, hardness tester (Wilson Hardness; ITW Test & Measurement, GmbH, Shanghai, China) was calibrated and set with a 0.8-mm blunt indenter to test specimens with 300 g load for 15 seconds. Each specimen was tested at 5 points and the average was calculated in Vickers hardness number (VHN). 15 21 The measurements of surface roughness were conducted using a noncontact profilometer (Contour Gt-K1 optical profiler, Bruker Nano, Tucson, Arizona, United States). A standard camera 20× was used to scan an area of 0.43 × 0.58 µm. Five scans were done at five different areas per specimen and then the average was calculated in µm. 15 21

### Statistical Analysis

Mean and standard deviations (SDs) were calculated and normality of distribution was verified with Wilk test. One-way analysis of variance (ANOVA) with Tukey's post hoc test was conducted to find out differences among test groups in flexural strength, surface roughness, and hardness. Two-way ANOVA was utilized to determine the influence of materials and immersion solutions. All statistical analyses were made with SPSS (v.26, IBM, Armonk, New York, United States).

## Results

Mean values, SDs, and significance for all tested properties between groups are displayed in Tables 2 , 3 , 4 . Two-way ANOVA results for the combined effect of materials and solutions were presented in Table 5 . Significant differences of flexural strength between different resin materials were found based on ANOVA analysis ( *p* < 0.001). After immersion in different salivary pH solutions for 90 days, the flexural strength values were adversely affected. Regarding the immersion groups, the highest flexural value (93.96 ± 3.18 MPa) was reported for IvoCad after immersion in DW while the lowest value (60.43 ± 2.66 MPa) was found in NextDent after immersion in 5.7 pH saliva. Milled specimens generally had superior flexural strength, followed by heat-polymerized specimens, while 3D-printed specimens had the lowest values, regardless of the immersion solution used ( *p* < 0.001) ( Table 2 ).

**Table 2:** Mean (SD) and significances between tested groups regarding saliva pH effect on flexural strength (MPa)

**pH**	**Conventional**	**AvaDent**	**IvoCad**	**FormLabs**	**NextDent**	***p***
T _0_	75.82 (1.99)	98.58 (2.92) ^A^	98.1 (3.51) ^a,A^	71.02 (1.8) ^B^	69.29 (2.66) ^B^	˂ 0.001*
5.7	67.75 (2.13) ^a^	88.73 (2.78) ^a,A^	87.4 (2.81) ^b,A^	62.74 (1.78) ^a,B^	60.43 (2.66) ^a,B^	˂ 0.001*
7.0	70.96 (3.35) ^b^	89.72 (1.86) ^a,b,A^	90.97 (3.7) ^b,c,A^	65.0 (2.18) ^a,b,B^	61.86 (3.11) ^a,b,B^	˂ 0.001*
8.3	70.82 (1.78) ^a,b^	91.56 (1.97) ^a,b^	81.35 (4.94)	65.19 (2.54) ^a,b,A^	63.79 (2.43) ^b,A^	˂ 0.001*
DW	72.54 (2.97) ^b^	92.65 (2.80) ^b,A^	93.96 (3.18) ^a,c,A^	65.88 (1.55) ^b,B^	64.50 (1.77) ^b,B^	˂ 0.001*
*p*	˂ 0.001*	˂ 0.001*	˂ 0.001*	˂ 0.001*	˂ 0.001*	

Abbreviations: DW, distilled water; SD. Standard deviation.

Note: Same small letter indicates statistically insignificant difference comparing solutions (columns), while same capital letter indicates statistically insignificant difference in means comparing materials (rows).

*Statistically significant at 0.05 level of significance.

**Table 3:** Mean (SD) and significances between tested groups regarding saliva pH effect on hardness (VHN)

**pH**	**Conventional**	**AvaDent**	**IvoCad**	**FormLabs**	**NextDent**	***p***
T _0_	52.23 (2.24)	56.56 (2.30) ^a^	47.50 (2.22) ^a^	41.81 (2.03) ^a^	33.83 (2.49)	˂ 0.001*
5.7	48.34 (4.51) ^a,A,B^	51.85 (3.65) ^a,A^	41.61 (2.75) ^b,c,B^	31.25 (10.15) ^b,C^	25.95 (2.85) ^a,C^	˂ 0.001*
7.0	44.88 (1.80) ^a,A,B^	53.71 (6.39) ^a,A^	40.60 (2.98) ^b^	38.91 (11.17) ^a,b,B,C^	26.98 (2.84) ^a,C^	˂ 0.001*
8.3	46.20 (1.92) ^a,A^	52.45 (6.90) ^a^	40.43 (2.85) ^b,A,B^	38.72 (7.53) ^a,b,B^	24.91 (2.13) ^a^	˂ 0.001*
DW	48.01 (2.34) ^a^	53.76 (1.60) ^a^	53.76 (1.60) ^a,c^	36.93 (2.51) ^a,b^	26.12 (3.29) ^a^	˂ 0.001*
*p*	˂ 0.001*	0.217	˂ 0.001*	˂ 0.001*	˂ 0.001*	

Abbreviations: DW, distilled water; SD. Standard deviation.

Note: Same small letter indicates statistically insignificant difference comparing solutions (columns) while same capital letter indicates statistically insignificant difference in means comparing materials (rows).

**Table 4:** Mean (SD) and significances between tested groups regarding saliva pH effect on the surface roughness (µm)

**pH**	**Conventional**	**AvaDent**	**IvoCad**	**FormLabs**	**NextDent**	***p***
T _0_	0.446 (0.061) ^a^	0.707 (0.059) ^a^	0.571 (0.054) ^a^	0.908 (0.044)	1.109 (0.094) ^a^	˂ 0.001*
5.7	0.809 (0.295) ^b,A^	1.206 (0.317) ^b,B^	0.757 (0.218) ^a,b,A^	1.942 (0.296) ^a^	1.438 (0.15) ^a,b,B^	˂ 0.001*
7.0	0.704 (0.303) ^a,b,A^	1.201 (0.465) ^b,B^	0.834 (0.228) ^b,A,B^	1.934 (0.351) ^a,C^	1.79 (0.444) ^b,c,C^	˂ 0.001*
8.3	1.368 (0.307) ^A^	1.328 (0.146) ^b,A^	1.436 (0.286) ^A^	1.368 (0.307) ^b,A^	2.263 (0.644) ^c^	˂ 0.001*
DW	0.594 (0.064) ^a,b,A^	0.854 (0.082) ^a,B^	0.757 (0.124) ^a,b,A,B^	1.454 (0.35) ^b^	1.2 (0.226) ^a^	˂ 0.001*
*p*	˂ 0.001*	˂ 0.001*	˂ 0.001*	˂ 0.001*	˂ 0.001*	

Abbreviations: DW, distilled water; SD. Standard deviation.

Note: Same small letter indicates statistically insignificant difference comparing solutions (columns) while same capital letter indicates statistically insignificant difference in means comparing materials (rows).

*Statistically significant at 0.05 level of significance.

**Table 5:** Two-way ANOVA analysis for combined effect of material and immersion solution on flexural strength, surface roughness, and hardness

**Property**	**Source**	**Sum of square**	**df**	**Mean square**	***F*** **-value**	***p*** **-Value**
Flexural strength	Material	36301.9	4	9075.5	1317.9	0.000 a
	Solution	2658.3	4	664.6	96.5	0.000 a
	Material*Solution	933.9	16	58.4	8.5	0.000 a
	Error	1549.4	225	6.9		
	Total	41443.5	249			
Surface roughness	Material	25.1	4	6.3	90.6	0.000 a
	Solution	18.7	4	4.7	67.7	0.000 a
	Material*Solution	10.7	16	0.7	9.7	0.000 a
	Error	15.6	225	0.07		
	Total	70.1	249			
Hardness	Material	20039.4	4	5009.9	241.6	0.000 a
	Solution	1359.1	4	339.8	16.4	0.000 a
	Material*Solution	582.3	16	36.4	1.8	0.039 a
	Error	4664.9	225	20.7		
	Total	26645.8	249			

Abbreviations: ANOVA, analysis of variance; df, degrees of freedom.

a Statistically significant at 0.05 level of significance.

The representative SEM micrographs of 5.7, 7.0, and 8.3 pH groups: conventional (heat-polymerized), CAD/CAM milled (IvoCad and AvaDent), and 3D-printed (FormLabs and NextDent) denture base resins are shown in Fig. 1 . For 5.7 pH specimens, a smooth surface with small uniform steps on the fractured surface were seen for conventional specimen ( Fig. 1 , A1), while irregular surface with randomly distributed lamellae and flack-like appearance and patchy surface was observed for AvaDent and IvoCad specimens ( Fig. 1 , B1 and C1). For FormLabs, the surface features were changed from rough to smooth background with faint lamellae and steps along the surface (see Fig. 1 , D1). NextDent fractured resin showed irregular surface with absence of lamellae in addition to the presence of one sharp step along the fractured surface representing layering fracture ( Fig. 1 , E1). Based on SEM findings there were variations in surface topography in term of materials type and different salivary pH effect, especially the appearance of surface particles with increasing pH (see Fig. 1 , A3–E3, lower panel).

All means, SDs, and the level of significance for all groups regarding the hardness test are presented in Table 3 . When comparing the immersion groups, AvaDent and IvoCad had the highest surface hardness (53.76 ± 1.6 VHN) after being immersed in DW, while NextDent showed the lowest value (24.91 ± 2.13 VHN) after immersion in 8.3 pH saliva.

Means, SDs, and the *p* -values of all tested groups in regards to surface roughness are shown in Table 4 . Among the immersion groups, the NextDent group presented the highest surface roughness mean value (2.26 ± 0.64 µm) after being immersed in 8.3 pH saliva while the conventional resin group had the lowest surface roughness (0.59 ± 0.06 µm) after the immersion in DW. Heat-polymerized, IvoCad, and NextDent specimens showed higher surface roughness after immersion in 8.3 pH saliva ( *p* < 0.001), while AvaDent showed increase in surface roughness after immersion in 5.7, 7.0, and 8.3 pH solutions compared with DW immersion and baseline measurements ( *p* < 0.001). FormLabs specimens showed higher surface roughness in all immersion solutions compared with baseline values ( *p* < 0.001) with specimens immersed in 5.7 and 7.0 pH being the highest.

## Discussion

Salivary pH can vary during the day due to the consumption of certain foods and beverage; moreover, some medical conditions can affect the pH of saliva. The purpose of this study was to assess the influence of various salivary pH on the flexural strength, hardness, and surface roughness of CAD/CAM denture base materials. The results demonstrated differences in the flexural strength, hardness, and surface properties among different materials immersed in different pH solutions. Therefore, the hypothesis of the current study stating that different salivary pH may influence the flexural strength and surface properties of denture base materials was accepted.

PMMA denture base resins gradually absorb water when immersed in fluids for long time. This is due to resin molecules' nature and polarity of resin properties. 22 The absorbed water displace the polymer chains, penetrate the polymer network of resin, and reduce the intermolecular force creating internal stress and cracks formation which results in weak mechanical properties. 23 24 Also, the residual monomer released from acrylic resins when immersed in water adversely affects the mechanical properties. Jagini et al confirmed the water sorption effect on flexural strength by immersing denture base resins in water and saliva at different intervals (15, 30, 60, 120 days) and reported that the flexural strength showed obvious decrease from 15 to 120 days regardless of the type of resin used. 25 The findings of Jagini et al's study was in accordance with the results of the current study which showed that water and saliva immersion resulted in decreased flexural strength regardless of the material type. In agreement with another study investigating the effect of water storage for 7 and 21 days on denture base resin with fiber reinforcement found that water storage decreased the flexural strength of unmodified and fiber-modified denture base resin. 26 This decrease may be related to water sorption effects since water can act as a plasticizers and thus reducing mechanical properties. 15 25 26 27

The effect of water sorption which was higher in 3D-printed specimens was in agreement with the results of previous study that showed high water sorption of 3D-printed resins compared with conventional heat-polymerized acrylic resin. 28 This may be attributed to the polymerization process, compared with heat polymerization which produces more dense specimens under pressure, high temperature, and with lower level of residual monomer. 29 In addition, monomer reactivity of 3D-printed resins has been reported to produce lower degree of conversion. 27 30 Layering nature of 3D printing may also be considered to cause decreased flexural strength values, as the bonding between successive printed layers is relatively week. 15

Milled denture base materials exhibited the highest flexural strength and hardness, and the lowest surface roughness compared with other tested groups. This might be due to the fabrication processes as specimens are milled from a prepolymerized disc. These discs are fabricated under pressure and at high temperatures resulting in high degree of monomer conversion. 31 32

After immersion in 5.7 pH of artificial saliva, IvoCad and AvaDent exhibited the highest flexural strength, followed by conventionally processed specimens, whereas FormLabs and NextDent had the lowest flexural strength values. All specimens had decreased flexural strength when compared with baseline values. These findings propose that acidic oral environment decreases flexural strength, which is in agreement with previous studies. 33 34 Tuna et al 35 reported that residual monomer release could be higher in conditions associated with acidic saliva than neutral saliva, which could lead to reduction in the acrylic resin flexural strength.

Denture hardness values could be affected by water sorption of polymeric materials. 33 In the present study, milled denture base materials show the highest hardness value as immersed in different pH solutions, in comparison to other denture base materials, while 3D-printed specimens had significantly lower hardness values especially after immersion in acidic solution of 5.7 pH. This is comparable to the results observed by Jafari et al, 36 who found that the commonly consumed beverages with low pH could result in hardness reduction of the acrylic base material. 36 Al-Otaibi et al 37 demonstrated that immersing different denture base materials in acidic solutions had the highest amount of monomer leakage, which directly affect surface properties including hardness. Another study reported a decrease in microhardness of the resin materials after specimens' storage in ethanol and heptane solutions with various proportions used to mimic the human diets. 34

The acceptable clinical value of surface roughness of dental materials in the oral cavity is < 0.2 µm. 38 39 All specimens tested in this study showed an increase in surface roughness after immersion in different salivary pH, compared with baseline. This is in agreement with Alfadda et al, 40 that reported a significant increase in the surface roughness of heat-polymerized acrylic resins after immersion in basic and neutral pH solutions. This could be referred to the impact of changes in pH, which could accelerate polymers degradation. 9 Release of hydroxyl ions that accelerate material degradation was noted at the basic pH solutions, leading to a remarkable surface roughness increase. 40 41 In addition, all specimens exhibited a higher surface roughness when the pH was more acidic as supported by previous studies 6 10 which indicated that the roughness of acrylic resin surfaces was increased when immersed in acidic solutions. However, milled groups had the least change in surface roughness in comparison to conventional and 3D-printed resins. 42

Specimens in this study were subjected to repeated cycles of immersion in different pH solutions to better simulate pH changes in the oral cavity. This helped in evaluating the influence of acidic, basic, and neutral pH on surface properties as well as flexural strength. The limitation of this study is it's in vitro nature, which requires careful interpretation of the results into clinical practice. Oral fluids typically contain enzymes, minerals, and other contents which were not considered in the methodology of the current study. Challenges in the oral cavity include simultaneous thermal and pH cycles, which may affect the reported results. Another limitation related to specimens configurations is it is fabricated in a bar shape and not in full dentures. Therefore, further investigations are required to test behaviors of materials after long-term cycling of immersion as well as to simulate oral environment.

## Conclusion

Different salivary pH had a negative influence on the flexural strength and surface properties of denture base materials. Milled denture bases showed higher flexural strength, hardness, and less surface roughness compared with conventional and 3D-printed resins. With different salivary pH, milled and conventional denture bases exhibited clinically acceptable flexural strength and hardness values, while 3D-printed specimens showed low mechanical properties which necessitate further investigations to improve its surface and mechanical properties.
